# Endometrial Cancer Incidence in Endometriosis and Adenomyosis

**DOI:** 10.3390/cancers13184592

**Published:** 2021-09-13

**Authors:** Marjolein Hermens, Anne M. van Altena, Iris Velthuis, Danielle C. M. van de Laar, Johan Bulten, Huib A. A. M. van Vliet, Albert G. Siebers, Ruud L. M. Bekkers

**Affiliations:** 1 Department of Obstetrics and Gynaecology, Catharina Hospital, 5623 EJ Eindhoven, The Netherlands; marjolein.hermens@radboudumc.nl (M.H.); dcmvandelaar@gmail.com (D.C.M.v.d.L.); Huib.v.Vliet@catharinaziekenhuis.nl (H.A.A.M.v.V.); 2 Department of Obstetrics & Gynaecology, Radboud University Medical Center, 6525 GA Nijmegen, The Netherlands; Anne.vanAltena@radboudumc.nl (A.M.v.A.); iris.velthuis@hotmail.com (I.V.); 3 Faculty of Health, Medicine and Life Sciences (FHML), Maastricht University, 6229 ER Maastricht, The Netherlands; 4 Department of Pathology, Radboud University Medical Center, 6525 GA Nijmegen, The Netherlands; Hans.Bulten@radboudumc.nl; 5 Department of Obstetrics and Gynaecology, University Hospital Ghent, 9000 Ghent, Belgium; 6 PALGA, The Nationwide Network and Registry of Histo- and Cytopathology in The Netherlands, 3991 SZ Houten, The Netherlands; Bert.Siebers@radboudumc.nl; 7 GROW School for Oncology and Developmental Biology, Maastricht University Medical Center, 6229 ER Maastricht, The Netherlands

**Keywords:** endometrial cancer, endometriosis, adenomyosis, risk

## Abstract

**Simple Summary:**

Previous research shows that women with endometriosis and adenomyosis have an increased ovarian cancer risk. However, it is unclear whether these women have an increased risk of developing uterine cancer. This information is of key importance to women with endometriosis or adenomyosis. Therefore, this study aims to assess the uterine cancer risk in women with endometriosis or adenomyosis in a large population.

**Abstract:**

Women with histologically proven endometriosis/adenomyosis have an increased risk of ovarian cancer. Small studies show conflicting results on the endometrial cancer risk in women with endometriosis/adenomyosis. Therefore, we assessed the incidence of endometrial cancer in women with histologically proven endometriosis or adenomyosis. We performed a population-based retrospective cohort study of 129,862 women with histologically proven endometriosis/adenomyosis, matched with 132,700 women with a nevus selected from the Dutch pathology registry between 1990 and 2015. Histology results for endometrial cancer were retrieved. Crude and age-adjusted odds ratios for endometrial cancer were estimated. In the endometriosis/adenomyosis group, 1827 (1.4%) women had a histological report on endometrial cancer, and in the nevus group, 771 (0.6%) women. The age-adjusted OR for endometrial cancer was 2.58 (95%CI 2.37–2.81). After excluding the first year of follow-up, the age-adjusted OR was 0.76 (95%CI 0.63–0.92), indicating that endometrial cancer is most often found at time of histological diagnosis of endometriosis/adenomyosis. In around 20% of the endometrial cancer cases, the endometrial cancer was not recognized until after hysterectomy. Of these women, 35% had no prior (micro)curettage or biopsy. This study shows an increased incidence of endometrial cancer in women with histologically proven endometriosis and adenomyosis.

## 1. Introduction

Endometriosis and adenomyosis are prevalent benign gynecological conditions in which endometrial-like glands and stroma are present outside the uterine cavity or in the myometrium, respectively [1,2,3,4]. Endometriosis and adenomyosis share characteristics with malignant tissue including tissue invasion, increased proliferative capability, induction of angiogenesis, the ability to evade apoptosis, and the ability to develop local and distant foci [5,6].

In 2018, around 380,000 women were diagnosed with endometrial cancer worldwide [7], and it is the most common gynecological cancer in developed countries [8]. Endometrial cancer prognosis is relatively good, as is it often found in the early stage [8]. The five most common histopathological subtypes are endometrioid, clear-cell, serous, mucinous endometrial cancer and adenocarcinoma not otherwise specified (NOS) [8].

Several studies have shown that endometriosis is associated with an increased risk of ovarian cancer, specifically endometrioid and clear cell ovarian subtypes [9,10,11]. However, contradictory evidence exists as to whether endometriosis and adenomyosis are associated with endometrial cancer [11,12,13,14,15]. Additionally, most studies included women with clinical or surgical endometriosis/adenomyosis, whereas histological diagnosis is still considered the gold standard [2]. Furthermore, studies on adenomyosis and endometrial cancer included small samples sizes [13,14,15].

Given the contradictory results and scarce evidence, especially for adenomyosis, larger epidemiological studies are warranted to elucidate the possible association between endometriosis/adenomyosis and endometrial cancer. Therefore, the objective of this study is to assess the incidence of endometrial cancer in women with histologically proven endometriosis or adenomyosis, and to determine whether there is a specific relationship with certain histological endometrial cancer subgroups.

## 2. Results

### 2.1. Cohort Characteristics

**Figure 1:**
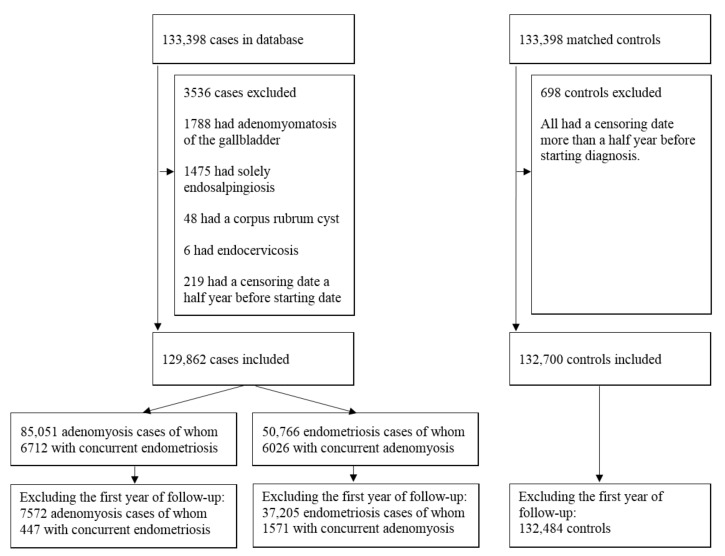
Consort flow diagram of the study population of cases and controls.

A total of 133,398 women with histologically detected endometriosis between 1990–2015 and 547,924 women with a benign dermal nevus were identified. Frequency matching resulted in a total of 266,796 women, 133,398 in both cohorts. In total, 3317 women were excluded in the endometriosis/adenomyosis cohort, as 1788 had adenomyomatosis of the gallbladder, 1475 had solely endosalpingiosis, 48 had a corpus rubrum cyst without endometriosis/adenomyosis, and six women had endocervicosis. Additionally, women with a censoring date more than half a year before start diagnosis were excluded, which resulted in 132,700 women in the nevus cohort and 129,862 in the endometriosis/adenomyosis cohort. For the separate analysis of endometriosis and adenomyosis, a total of 85,051 women were eligible in the adenomyosis cohort and 50,766 in the endometriosis cohort (Figure 1). In the adenomyosis group, 6712 women had concurrent endometriosis, and in the endometriosis group, 6026 women had concurrent adenomyosis. The number of women with endometriosis combined with adenomyosis varied compared to the number of women with adenomyosis combined with endometriosis due to differences in date of the chosen starting diagnosis (endometriosis versus adenomyosis), and therefore differences in exposure years and subsequent exclusions exist.

The median age at endometriosis/adenomyosis diagnosis was 44 years, with an inter quartile range (IQR) of 38–50 years, whereas this was 45 years (IQR 38–51) in the nevus cohort, *p* = 0.09. Median follow-up in the endometriosis/adenomyosis cohort was zero years (range 0–27 years) and 16 years (range 0–27) in the nevus cohort, *p* < 0.001. This resulted in 606,083 and 2,029,597 person-years per cohort, respectively. The data for the endometriosis and adenomyosis, respectively, are reported in Table 1 and Table S1.

After excluding the first year of follow-up 37,205, 7572 and 132,484 women remained in the endometriosis, adenomyosis and nevus cohorts, respectively. This large number of exclusions in the second analysis was mainly due to censoring because of hysterectomies. A total of 15,695 (30.9%) women eventually underwent a hysterectomy in the endometriosis cohort, 77,993 (91.7%) women in the adenomyosis cohort, and 1982 (1.5%) women in the nevus cohort. The remaining women in the adenomyosis cohort mostly had adenomyomectomies. Several women had their endometrial cancer diagnosis at time of hysterectomy; however, without any previous histological diagnosis of endometrial cancer in a (micro)curettage or smear. However, most of these women had a previous (micro)curettage with endometrial hyperplasia, atypia or endometrial polyp (Table 2) or a smear showing atypical endometrial cells. Strikingly, 38% in the endometriosis cohort, 33% in the adenomyosis cohort, and 28% in the nevus cohort had no previous endometrial sampling or smear at all.

### 2.2. Endometrial Cancer

We detected 1827 endometrial cancer cases in the endometriosis/adenomyosis group and 771 endometrial cancer cases in the nevus group (Table 1). Age at endometrial cancer diagnosis in the endometriosis cohort (59 years) was significantly different from the adenomyosis (61 years) and nevus cohort (62 years), *p* < 0.001. There was no difference in age at endometrial cancer diagnosis between the adenomyosis and nevus cohort (Table 1). In the metachronous selection time from inclusion to endometrial cancer diagnosis was 10 years (IQR 6–16) in the adenomyosis cohort, 13 years (IQR 7–18) in the endometriosis cohort, and 13 years (IQR 8–18) in the nevus cohort (not significant).

### 2.3. Odds Ratio of Endometrial Cancer

Including the whole follow-up period resulted in a crude OR of 2.44 (95%CI 2.24–2.66) and age-adjusted OR of 2.58 (95%CI 2.37–2.81) when comparing the endometriosis/adenomyosis cohort with the nevus cohort. The OR was highest for adenocarcinomas NOS. The crude and age-adjusted ORs for all endometrial cancer subtypes for the endometriosis/adenomyosis cohort, the nevus cohort and the separated endometriosis and adenomyosis cohort are shown in Table 3.

Excluding the first year of follow-up (metachronous group) resulted in a crude OR of 0.59 (95%CI 0.49–0.70) and age-adjusted OR of 0.76 (95%CI 0.63–0.92). The Ors of the endometriosis group were similar to the overall group with a crude OR of 0.48 (95%CI 0.39–0.59) and age-adjusted OR of 0.65 (0.52–0.81). The Ors in the adenomyosis group were higher, as the crude OR was 1.11 (95%CI 0.82–1.50) and the age-adjusted OR was 1.11 (95%CI 0.82–1.50) for all endometrial cancer cases combined. The ORs with respect to endometrial cancer subtype are stated in Table 3. Sensitivity analysis excluding women with both endometriosis and adenomyosis did not result in significantly different ORs.

### 2.4. Histological Distribution of Endometrial Cancer Subtypes

Table 4 shows the histological distribution of the endometrial cancer subtypes. In general, there were relatively fewer women with endometrioid endometrial cancer in all the endometriosis/adenomyosis cohorts and more women with clear cell endometrial cancer or adenocarcinomas NOS. In the metachronous group the histological distribution in the cohorts was similar. A lot of cases in the endometriosis/adenomyosis cohort were assigned to the adenocarcinoma NOS due to lacking or inconclusive information. Most of these cases, however, were well differentiated tumors without any report of histological subtype.

## 3. Discussion

### 3.1. Principal Findings

This large nationwide cohort study observed an increased association between endometriosis/adenomyosis and endometrial cancer with an age-adjusted OR of 2.58 (95%CI 2.37–2.81). We found the highest ORs for clear cell endometrial cancer subtype (OR 30.31 95%CI 9.83–93.51) and adenocarcinoma NOS (OR 79.61 95%CI 61.70–102.72). The histological diagnosis of endometriosis/adenomyosis and endometrial cancer was synchronously diagnosed in most women. After excluding the first year of follow-up there was a substantial reduction of endometrial cancer cases in the endometriosis/adenomyosis cohort and as a consequence, the observed ORs for endometrial cancer in the endometriosis/adenomyosis cohort showed no increased association.

### 3.2. Results of the Study in the Context of Other Observations

A recent large meta-analysis showed a relative risk of 1.23 (95%CI 0.97–1.57) for endometrial cancer in women with solely endometriosis, whereas we found an age-adjusted OR of 2.63 (95%CI 2.35–2.95) [11]. The studies included in this meta-analysis had a high level of heterogeneity and mostly used self-reported or clinically diagnosed endometriosis instead of histologically diagnosed endometriosis. In addition, two nationwide studies form Finland and Scotland, including women with surgically confirmed endometriosis, found no increased risk of endometrial cancer [16,17]. These studies included women at a younger age compared to our study, but with similar follow-up time, which could possibly explain the lower endometrial cancer incidence in these studies in general. Moreover, our endometriosis cohort with histologically diagnosed endometriosis might have more severe disease with a potentially different risk profile.

In the adenomyosis cohort we found an age-adjusted OR for endometrial cancer of 2.63 (95%CI 2.40–2.87) and an age-adjusted OR of 1.11 (95%CI 0.82–1.50) after excluding the first year of follow-up. Kok et al. [15] found a similar adjusted hazard ratio of 4.38 (95%CI 1.22–15.72) for endometrial cancer in women with mostly surgically diagnosed adenomyosis with preserved uterus and ovaries at time of clinical diagnosis. Similarly, the meta-analysis by Raffone et al. showed that the prevalence of adenomyosis in women with diagnosed endometrial cancer was similar to the prevalence reported in hysterectomies for other gynecological conditions [13]. The included studies, however, did not assess a control group of women without endometrial cancer, and therefore a direct comparison was not possible.

Due to the nature of our study, the women in the endometriosis/adenomyosis cohort more frequently had a hysterectomy as compared to the nevus cohort. We hypothesize that the women who underwent a hysterectomy might have had a higher endometriosis/adenomyosis disease burden or clinically showed no adequate response to hormonal treatment, possibly resulting in a higher risk for endometrial cancer. In contrast, the women with a better response to hormonal treatment might have been on hormonal therapy for longer and therefore might have had a decreased risk of developing endometrial cancer, as in general, the use of oral contraceptives causes a decrease in the risk of endometrial cancer by about 50% [18], and could therefore explain the lower risk found in the endometriosis/adenomyosis cohort with more than a year of follow-up.

Strikingly, of all women with endometrial cancer and a hysterectomy, roughly 20% had no endometrial cancer diagnosis before hysterectomy, and of this group around 35% had no previous endometrial sampling within a year of endometrial cancer diagnosis at all. These women were possibly being treated for benign uterine diseases, but unexpectedly had endometrial cancer diagnosed. We therefore recommend considering endometrial sampling before a hysterectomy, especially in the case of severe endometriosis/adenomyosis complaints, as knowing the malignant status preoperatively will often have consequences for the surgical procedure, i.e., staging.

### 3.3. Endometrial Cancer Subtypes

Endometrial cancer subtypes have rarely been evaluated in previous studies. One study showed a stronger association for type I endometrial cancers (endometrioid, mucinous endometrial cancer and adenocarcinoma NOS) [19], which is partially in accordance with our findings. The reason for the high number of adenocarcinoma NOS cases in the endometriosis/adenomyosis cohort is not clear. Most of the adenocarcinoma NOS were low-grade tumors without any specific report of histological subtype. Unfortunately, it was not possible to review the samples, but we hypothesize that these cases were mostly well-differentiated endometrioid endometrial cancers, as clear cell and serous endometrial cancer are per definition classified as high-grade tumors [20].

Type I endometrial cancers are commonly associated with a relatively good prognosis [8]. A recent meta-analysis showed that women with adenomyosis and endometrial cancer had longer overall survival when compared to women with endometrial cancer without adenomyosis [21]. However, in this study it was not possible to calculate multivariate hazard ratios. Our study group recently performed a nationwide cohort study comparing survival in women with endometrial cancer with or without endometriosis/adenomyosis [22]. In this study, we found increased overall survival after endometrial cancer diagnosis in women with endometriosis/adenomyosis. After correction for confounders like age, stage, grade and histological subtype no increased survival was found.

### 3.4. Age at Endometrial Cancer Diagnosis and Combined Endometriosis and Adenomyosis

In the metachronous analysis, women in the endometriosis cohort were younger (56 years) at endometrial cancer diagnosis when compared to the adenomyosis cohort (64 years) and the nevus cohort (62 years). The average age at endometrial cancer diagnosis in the Netherlands is 67 years [23]. The lower endometrial cancer age in the endometriosis cohort could be explained by endometriosis being a disease in young fertile women. These women are significantly younger at inclusion (36 years) when compared to the women with adenomyosis (45 years) or a nevus (45 years). The median follow-up in the endometriosis cohort is 12 years, which means that the average woman in the endometriosis cohort is 48 years at end of study follow-up, and therefore, that a large number of women in the endometriosis cohort might not have reached the average Dutch endometrial cancer age.

In our study, 6712 (7.9%) women in the endometriosis cohort had concurrent adenomyosis and in the endometriosis cohort 6026 (11.9%). This is in line with previously reported studies reporting endometriosis incidences between 3–18% in women with adenomyosis [24]. Excluding the cases with both endometriosis and adenomyosis did not alter the results.

### 3.5. Possible Key Factors in the Malignant Transformation of Endometriosis/Adenomyosis

Endometriosis and adenomyosis are both estrogen-dependent entities; additionally, type I endometrial cancers are associated with increased estrogen levels [25,26]. When looking into the hypothesis of the malignant transformation of endometriosis/adenomyosis, there might be a role for the immune system. In both endometriosis and adenomyosis, the immune system seems to be more active [27,28]. As the immune system also plays an important factor in carcinogenesis by tumor initiation, promotion and progression [29], an activated immune system in endometriosis/adenomyosis might play a key role in the malignant transformation of these diseases. However, additional studies are needed to test this hypothesis.

Studies on the possible malignant transition of adenomyosis are scarce. One study on molecular changes in adenomyosis showed upregulated Kirsten rat sarcoma virus (KRAS) genes and reduced Phosphatase and tensin homolog (PTEN) expression in adenomyosis [30]. Furthermore, the process of epithelial-to-mesenchymal transition (EMT) seems to be crucial in the development of adenomyosis but also plays an important role in carcinogenesis [31,32]. Several studies have shown that changes in genes like AT-rich interactive domain-containing protein 1A (ARID1A), PTEN, KRAS, phosphatidylinositol-4,5-bisphosphate 3-kinase, catalytic subunit alpha (PIK3CA) and Serine/threonine-protein phosphatase 2A 65 kDa regulatory subunit A alpha isoform (PPP2R1A) are present in women with endometriosis, but these are also known cancer-driving mutations involved in endometrial cancer carcinogenesis [33,34]. However, cancer-associated mutations were also found in endometriotic lesions without concurrent cancer, in particular in deep infiltrating lesions which are rarely associated with cancer development [35]. It remains unclear whether these mutations are key in malignant transformation of endometriosis/adenomyosis. Nonetheless, identification of possible driver mutations in endometriosis/adenomyosis samples might help in the future to identify women at risk for developing endometrial cancer.

### 3.6. Strengths and Limitations

The strength of this study is that it is a large nationwide study in which we only included women with histologically proven endometriosis or adenomyosis, which is still considered the gold standard for these diagnoses. However, using a histological database can also be considered a limitation, as no clinical data were available. As women with endometriosis/adenomyosis often have other known risk factors for cancer development, studies adjusting for these possible confounders are warranted. Due to the nature of our database, it was not possible to correctly differentiate endometriosis subtypes. Furthermore, women in the nevus cohort could have had a clinical diagnosis of endometriosis/adenomyosis without histological confirmation. Another limitation of our study is the high number of hysterectomies in the adenomyosis cohort, and consequently the low number of exposure years. We therefore performed logistic regression analysis to calculate odds ratios. Additionally, it is not known whether the women in the nevus cohort had had a hysterectomy before start of the study, but since median age at inclusion was 45 years, a high rate of hysterectomies before start of study seems unlikely. Previously, two studies showed a slightly increased incidence (2–3% increase) of benign dermal nevi in women with laparoscopic confirmed endometriosis [36,37]. To our knowledge, no association between ovarian cancer and nevi exists; we therefore believe the effect of this association on our results is limited. Moreover, this study is prone to detection bias, and therefore we performed a second analysis excluding the first year of follow-up. Lastly, PALGA uses identification codes based on the first eight letters of the family name and birth date, therefore results from different women may have been combined. We believe the effect of this is minimized by using a large control cohort with the same risk of merged cases.

## 4. Future

To develop preventive strategies, future studies should focus on detection of women at risk for endometrial cancer in the group of women with endometriosis/adenomyosis. Although endometriosis/adenomyosis and endometrial cancer share several risk factors, most women with endometriosis/adenomyosis do not develop endometrial cancer. It is therefore important to identify specific risk factors for endometrial cancer in these women.

In recent years, advancements in magnetic resonance imaging (MRI) have enabled reliable non-invasive methods for diagnosing adenomyosis [38]. To ensure longer follow-up, future studies should consider using strictly defined MRI criteria for the diagnosis of endometriosis or adenomyosis.

## 5. Material and Methods

### 5.1. Study Population and Design

Previously, we selected all women with histological codes for “endometriosis” and “adenomyosis” between 1990 and 2015 from the Dutch nationwide registry of histopathology and cytopathology (PALGA, Houten, The Netherlands) [7]. In the initial study, these women were randomly frequency matched with women diagnosed with a benign dermal nevus, but with no histological endometriosis or adenomyosis diagnosis from the same database. We chose women with a histologically diagnosed nevus as a control group, because it can be diagnosed in all women of all ages.

Histological reports for endometrial cancer between 1 January 1990 and 1 July 2017 were retrieved for all women. Each endometrial cancer case was assigned to an endometrial cancer subtype, i.e., endometrioid, clear cell, serous, mucinous, and adenocarcinoma not otherwise specified (NOS). If the endometrial cancer subtype was not reported unequivocally, the case was assigned to the adenocarcinoma NOS subtype. All indistinct endometrial cancer diagnoses were discussed with a second reviewer and consensus was reached. We censored women after endometrial cancer diagnosis, hysterectomy, autopsy, or end of follow-up. Women who had a censoring date six months or more before the start diagnosis were considered ineligible.

### 5.2. Statistical Analysis

We analyzed the endometriosis/adenomyosis cohort as a whole and separately, with each woman in the cases cohort being assigned to the endometriosis or adenomyosis cohort. If a woman had both diseases, she was included in both cohorts.

As a subgroup analysis, we excluded all women with less than one person-year at risk to account for detection bias. The remaining cases were classified as the metachronous group. For each cohort the number of endometrial cancer cases was determined.

We aimed to calculated incidence rates and incidence rate ratios, but due to a very low number of exposure years, especially in the adenomyosis cohort, the calculated results were considered less reliable, as ‘exposure years’ is a key variable in calculating the IRR. Therefore, we decided to calculate crude and age-adjusted odds ratios by logistic regression analysis. We performed a sensitivity analysis excluding cases with both endometriosis and adenomyosis.

Chi-square tests were used to compare the distribution of endometrial cancer subtypes between the endometriosis/adenomyosis cohort and the nevus cohort. All statistical analyses were performed with SPSS version 25.0.0.1 for Windows (SPSS, Chicago, IL, USA) and STATA v15.1 (StataCorp LLC, College Station, TX, USA).

## 6. Conclusions

In conclusion, we found an increased incidence of endometrial cancer in both women with endometriosis and adenomyosis. After excluding the first year of follow-up no increased incidence was found, which might suggest that this increased incidence is largest for women with a more extensive endometriosis/adenomyosis disease burden or a poorer response to hormonal treatment. Future studies are warranted to identify women with endometriosis or adenomyosis at risk of developing endometrial cancer and to develop a risk stratification for cancer development. Additionally, clinicians should consider endometrial sampling before hysterectomy in cases of endometriosis or adenomyosis, as a large proportion of endometrial cancer was found in women without prior endometrial sampling.

## Figures and Tables

**Table 1:** Study characteristics—per endometriosis, adenomyosis and nevus group. Data are in numbers, percentages (%), years, median (IQR). Summed up numbers of endometriosis and adenomyosis are larger than the cases total because there were cases diagnosed with both endometriosis and adenomyosis. Metachronous group is defined as endometriosis, adenomyosis or nevus diagnosis at least a year before censoring date (autopsy, hysterectomy, or endometrial cancer).

Characteristics	Endometriosis/Adenomyosis Combined	Endometriosis	Adenomyosis	Nevus
Total Group				
Included patients in analysis	129,862	50,766	85,051	132,700
Age at study inclusion (IQR)	44 (38–50) ^1^	39 (32–45) ^3^	47 (42–52) ^3^	45 (38–51)
Median inclusion year	2001 (1995–2008) ^1^	2002 (1996–2009) ^3^	2000 (1994–2007) ^3^	2001 (1995–2008)
Median follow up (range)	0 (0–27) ^2^	8 (0–27) ^3^	0 (0–27) ^3^	16 (0–27)
Person years	606,083	492,278	119,465	2,029,597
Hysterectomy (%)	88,112 (67.9%) ^2^	15,695 (30.9%) ^3^	77,993 (91.7%) ^3^	1982 (1.5%)
Number of endometrial cancer cases (%)	1827 (1.4%) ^2^	519 (1.0%) ^3^	1455 (1.7%) ^3^	771 (0.6%)
Age at diagnosis of endometrial cancer (IQR)	61 (55–69) ^1^	59 (52–67) ^3^	61 (55–69) ^4^	62 (56–68)
Metachronous group				
Included patients in analysis	44,377	37,205	7572	132,484
Age at study inclusion (IQR)	37 (31–44) ^2^	36 (30–42) ^3^	45 (40–50) ^3^	45 (38–51)
Median inclusion year	2003 (1996–2009) ^2^	2004 (1997–2009) ^3^	1999 (1994–2007) ^3^	2001 (1995–2008)
Median follow up (range)	13 (1–27) ^2^	12 (1–27) ^3^	17 (1–27) ^3^	16 (1–27)
Person years	605,716	492,074	119,294	2,029,570
Hysterectomy (%)	2679 (6.0%) ^2^	2155 (5.8%) ^3^	551 (7.3%) ^3^	1778 (1.3%)
Number of endometrial cancer cases (%)	143 (0.3%) ^2^	98 (0.3%) ^3^	46 (0.6%) ^4^	726 (0.5%)
Age at diagnosis of endometrial cancer (IQR)	59 (52–65) ^2^	56 (51–63) ^3^	64 (57–70) ^4^	62 (56–68)

^1^ Not significant when compared to the nevus cohort. ^2^ *p* < 0.001 when compared to the nevus cohort. ^3^ *p* < 0.001 when compared to the nevus cohort and the endometriosis or adenomyosis solely cohort. ^4^ Not significant when compared to the nevus cohort but *p* < 0.001 when compared to the endometriosis solely cohort.

**Table 2:** Observed number of hysterectomies of women with endometriosis, adenomyosis or a benign dermal nevus with respect to endometrial cancer or no endometrial cancer. Further specification of all women with endometrial cancer diagnosis at time of hysterectomy with information on previous endometrial tissue sampling ^1^.

Endometrial Cancer Versus No Endometrial Cancer	Endometriosis	Adenomyosis	Nevus
Endometrial cancer (*n* = 2413)	**480 (3.1%)**	**1408 (1.8%)**	**666 (33.6%)**
Endometrial cancer diagnosis at time of hysterectomy (*n* = 451)	**109 (22.7%)**	**275 (19.5%)**	**103 (15.5%)**
Prior endometrial hyperplasia, atypia or polyp in (micro)curettage or cervical smear (n = 274) ^2^	60 (55.0%)	165 (60.0%)	69 (67.0%)
Prior benign endometrium or invalid (micro)curettage or cervical smear (n = 30) ^2^	8 (7.3%)	20 (7.3%)	5 (4.9%)
No previous endometrial sampling (n = 147) ^2^	41 (37.6%)	90 (32.7%)	29 (28.2%)
Hysterectomy after endometrial cancer diagnosis (n = 1962)	**371 (77.3%)**	**1133 (80.5%)**	**563 (84.5%)**
No endometrial cancer (n = 87,680)	**15,215 (96.9%)**	**76,585 (98.2%)**	**1316 (66.4%)**
Total (n = 90,093)	**15,695 (100%)**	**77,993 (100%)**	**1982 (100%)**

^1^ Summed up numbers are larger than the combined total because there were cases with both endometriosis and adenomyosis. ^2^ Within the last 365 days.

**Table 3:** Observed number of endometrial cancers, crude odds ratios, and age-adjusted odds ratios of endometrial cancers of women with endometriosis/adenomyosis combined, endometriosis solely, or adenomyosis solely compared with women with a benign dermal nevus, per endometrial cancer subtype and overall. Data are in numbers, percentages (%) or incidence rate ratios. Summed up numbers of endometriosis and adenomyosis are larger than the cases total because there were cases diagnosed with both endometriosis and adenomyosis. Metachronous group is defined as endometriosis, adenomyosis or nevus diagnosis at least a year before censoring date (autopsy, hysterectomy, or endometrial cancer).

Endometrial Cancer Subtypes Per Cohort	Total Group			Metachronous Group		
	ON	Crude OR (95%CI)	Age-Adjusted OR (95%CI)	ON	Crude OR (95%CI)	Age-Adjusted OR (95%CI)
**Endometrioid**						
Cases combined	1118	1.98 (1.79–2.19)	2.06 (1.86–2.28)	114	0.61 (0.50–0.74)	0.77 (0.63–0.95)
Endometriosis	327	1.48 (1.29–1.70)	2.08 (1.80–2.39)	80	0.51 (0.40–0.65)	0.68 (0.53–0.86)
Adenomyosis	885	2.40 (2.16–2.67)	2.11 (1.90–2.34)	34	1.07 (0.76–1.51)	1.07 (0.75–1.51)
Nevus	579	ref	ref	557	ref	ref
**Clear cell**						
Cases combined	28	2.86 (1.39–5.89)	3.02 (1.47–6.23)	3	1.00 (0.27–3.68)	1.45 (0.38–5.56)
Endometriosis	5	1.31 (0.45–3.82)	2.15 (0.72–6.40)	2	0.79 (0.17–3.66)	1.25 (0.26–6.13)
Adenomyosis	24	3.75 (1.79–7.83)	3.25 (1.56–6.81)	1	1.94 (0.25–15.35)	1.99 (0.25–15.72)
Nevus	10	ref	ref	9	ref	ref
**Serous**						
Cases combined	98	2.09 (1.48–2.95)	2.20 (1.55–3.10)	5	0.35 (0.14–0.88)	0.50 (0.20–1.27)
Endometriosis	26	1.42 (0.88–2.28)	2.24 (1.37–3.64)	2	0.17 (0.04–0.68)	0.24 (0.06–1.02)
Adenomyosis	81	2.63 (1.84–3.77)	2.29 (1.60–3.27)	3	1.22 (0.38–3.94)	1.24 (0.38–3.99)
Nevus	48	ref	ref	43	ref	ref
**Mucinous**						
Cases combined	9	1.84 (0.62–5.49)	2.02 (0.68–6.04)	0	NA	NA
Endometriosis	6	3.14 (0.96–10.28)	5.90 (1.77–19.63)	0	NA	NA
Adenomyosis	5	1.56 (0.45–5.39)	1.36 (0.39–4.70)	0	NA	NA
Nevus	5	ref	ref	5	ref	ref
**Adenocarcinoma NOS**						
Cases combined	574	4.56 (3.77–5.52)	4.84 (3.99–5.86)	21	0.56 (0.35–0.89)	0.76 (0.47–1.23)
Endometriosis	155	3.15 (2.49–3.98)	5.21 (4.10–6.62)	14	0.45 (0.26–0.78)	0.64 (0.36–1.13)
Adenomyosis	460	5.59 (4.60–6.80)	4.89 (4.02–5.95)	8	1.25 (0.61–2.56)	1.26 (0.61–2.57)
Nevus	129	ref	ref	112	ref	ref
**All endometrial cancers**						
Cases combined	1827	2.44 (2.24–2.66)	2.58 (2.37–2.81)	143	0.59 (0.49–0.70)	0.76 (0.63–0.92)
Endometriosis	519	1.77 (1.58–1.98)	2.63 (2.35–2.95)	98	0.48 (0.39–0.59)	0.65 (0.52–0.81)
Adenomyosis	1455	2.98 (2.73–3.25)	2.63 (2.40–2.87)	46	1.11 (0.82–1.50)	1.11 (0.82–1.50)
Nevus	771	ref	ref	726	ref	ref

Logistic regression was used to calculate odds ratios. Abbreviations: ON = observed number, OR = Odds Ratio, NOS = Not otherwise specified, CI = confidence interval. Bold: histological endometrial cancer subtypes.

**Table 4:** Histological distribution with respect to cohort. Data are in numbers or percentages (%). Summed up numbers of endometriosis and adenomyosis are larger than the cases total because there were cases diagnosed with both endometriosis and adenomyosis. Metachronous group is defined as endometriosis, adenomyosis or nevus diagnosis at least a year before censoring date (autopsy, hysterectomy, or endometrial cancer).

Histological Type	Endometriosis/Adenomyosis Combined	Endometriosis	Adenomyosis	Nevus
**Total group ^1^**				
Endometrioid	1118 (61.2%)	327 (63.0%)	885 (60.8%)	579 (75.1%)
Clear cell	28 (1.5%)	5 (1.0%)	24 (1.6%)	10 (1.3%)
Serous	98 (5.4%)	26 (5.0%)	81 (5.6%)	48 (6.2%)
Mucinous	9 (0.5%)	6 (1.2%)	5 (0.3%)	5 (0.6%)
Adenocarcinoma NOS	574 (31.4%)	155 (29.9%)	460 (31.6%)	129 (16.7%)
Total	1827 (100%)	519 (100%)	1455 (100%)	771 (100%)
**Metachronous group ^2^**				
Endometrioid	114 (79.7%)	80 (81.6%)	34 (73.9%)	557 (76.7%)
Clear cell	3 (2.1%)	2 (2.0%)	1 (2.2%)	9 (1.2%)
Serous	5 (3.5%)	2 (2.0%)	3 (6.5%)	43 (5.9%)
Mucinous	0 (0%)	0 (0%)	0 (0%)	5 (0.7%)
Adenocarcinoma NOS	21 (14.7%)	14 (14.3%)	8 (17.4%)	112 (15.4%)
Total	143 (100%)	98 (100%)	46 (100%)	726 (100%)

^1^ Histological distribution is significantly different for all endometriosis/adenomyosis cohorts in the total group when compared to the nevus cohort. ^2^ Histological distribution is not significantly different when compared to the nevus cohorts.

## Data Availability

The data used in this study is available at PALGA. Requests can be used to retrieve excerpts from PALGA’s national database. One of PALGA’s consultants will supervise the request procedure. All requests are reviewed by the Scientific Council and the Privacy Committee.

## Supplementary Materials

The following are available online at https://www.mdpi.com/article/10.3390/cancers13184592/s1, Table S1: Inclusion characteristics of study population.
