# Supplementary material for: Endometrial Cancer Incidence in Endometriosis and Adenomyosis
Source: Cancers (Basel). 2021 Sep 13;13(18):4592. doi: 10.3390/cancers13184592 (PMC8464914; doi:10.3390/cancers13184592)
Supplement: Supplementary file 1 [file cancers-13-04592-s001.zip › cancers-1314014-supplementary.pdf]

Table S1. Inclusion characteristics of study population.

Age/Year	Endometriosis		Adenomyosis		Nevus	
	**Number of patients (%)**	**Person-years of follow-up**	**Number of patients (%)**	**Person-years of follow-up**	**Number of patients (%)**	**Person-years of follow-up**
Age cohort						
0-29	8,292 (16.3%)	113,348	524 (0.6%)	3,512	9,019 (6.8%)	133,340
30-34	9,394 (18.5%)	11,5085	2,714 (3.2%)	8,450	12,022 (9.1%)	178,608
35-39	9,478 (18.7%)	96,634	8,955 (10.5%)	16,832	17,715 (13.3%)	276,453
40-44	9,477 (18.7%)	77,490	19,048 (22.4%)	27,683	27,205 (20.5%)	429,170
45-49	7,452 (14.7%)	50,191	23,530 (27.7%)	30,080	29,580 (22.3%)	459,744
50-54	3,244 (6.4%)	19,562	13,978 (16.4%)	17,794	16,709 (12.6%)	253,632
55-59	1,309 (2.6%)	7,903	6,105 (7.2%)	7,145	7,413 (5.6%)	108,537
60-64	739 (1.5%)	4,002	3,651 (4.3%)	2,811	4,575 (3.4%)	650,14
65-69	592 (1.2%)	3,105	2,791 (3.3%)	2,104	3,517 (2.7%)	51,423
70-74	356 (0.7%)	2,044	1,906 (2.2%)	1,240	2,416 (1.8%)	35,546
75-79	242 (0.5%)	1,447	1,206 (1.4%)	1,014	1,586 (1.2%)	24,053
80+	188 (0.4%)	1,420	641 (0.8%)	800	943 (0.7%)	14,077
Missing	3 (0.0%)	47	2 (0.0%)	0	0 (0.0%)	0
Total	50,766 (100.0%)	492,278	85,051 (100.0%)	119,465	132,700 (100.0%)	2,029,597
Calendar period						
1990-1994	10,584 (20.8%)	146,229	21,606 (25.4%)	52,565	31,144 (23.5%)	767007
1995-1999	9,538 (18.8%)	121,852	18,374 (21.6%)	31,503	27,122 (20.4%)	536690
2000-2004	9,361 (18.4%)	101,535	15,807 (18.6%)	18,385	24,551 (18.5%)	363712
2005-2009	10,264 (20.2%)	80,762	14,794 (17.4%)	11,605	24,587 (18.5%)	241943
2010-2015	11,019 (21.7%)	41,900	14,470 (17.0%)	5,407	25,296 (19.1%)	120245
Total	50,766 (100.0%)	492,278	85,051 (100.0%)	119,465	132,700 (100.0%)	2,029,597

Data are in numbers, percentages (%) or years.
